# A maize landrace introgression library reveals a negative effect of root‐to‐shoot ratio on water‐use efficiency

**DOI:** 10.1002/tpg2.70036

**Published:** 2025-04-25

**Authors:** Giuseppe Sciara, Francesco Camerlengo, Claude Welcker, Llorenç Cabrera‐Bosquet, Antonin Grau, Maria Angela Cané, Riccardo Bovina, Francois Tardieu, Roberto Tuberosa, Silvio Salvi

**Affiliations:** ^1^ Department of Agricultural and Food Sciences University of Bologna Bologna Italy; ^2^ INRAE, LEPSE Université de Montpellier Montpellier France

## Abstract

Novel sources of genetic variability for water‐use efficiency (WUE) are needed in order to breed varieties more suitable to sustainable cropping systems. Here, a maize (*Zea mays* L.) introgression library of the landrace Gaspé Flint into the reference line B73 was characterized in high‐throughput phenotyping platforms, both in well‐watered and moderate water‐deficit conditions, for water use, WUE, and root and shoot growth. Traits heritability ranged from 0.77 to 0.93. The introgression of Gaspé Flint chromosome segments into the B73 genome significantly altered several traits. Some introgression lines exhibited a faster shoot biomass accumulation than B73, resulting in higher WUE at the expense of root growth. Quantitative trait loci (QTL) mapping identified seven major QTL clusters affecting shoot growth and WUE, two of which overlapped, with opposite effects, with QTLs for root biomass known to include root developmental genes. These results support the non‐intuitive hypothesis that reduced root‐to‐shoot ratio positively affects maize WUE.

## Core Ideas

- A maize introgression library was phenotyped for water use, water‐use efficiency, root and shoot growth.
- Quantitative trait loci (QTL) mapping identified seven major QTL clusters affecting shoot growth and water‐use efficiency.
- Gaspé Flint landrace confers early vigor QTL haplotypes with beneficial effect on water‐use efficiency.
- Reduced root‐to‐shoot ratio positively affects maize water‐use efficiency.

**Abbreviations**
IL: introgression library
QTL: quantitative trait loci
wd: water‐deficit
WUE: water‐use efficiency
ww: well‐watered

## 1 INTRODUCTION

In maize (*Zea* *mays* L.), growth maintenance under water deficit (wd) is a well‐recognized breeding target; however, its achievement is challenged by high physiological and genetic complexity, and by the inherent interaction between genotype, environmental conditions, and management practices (G × E × M), which reduces trait heritability especially under drought (Cooper et al., 2014; Messina et al., 2023; Tardieu et al., 2018). An important part of drought‐targeted breeding is the identification of traits affecting the responses of growth to wd and water‐use efficiency (WUE), which may show higher heritability than grain yield under stress conditions (Ziyomo & Bernardo, 2013), and understanding their genetic and molecular control (Cooper & Messina, 2023; Tuberosa, 2012). This is particularly crucial in maize where studies have shown that modern breeding, while able to overall increase yield under drought, failed to decrease sensitivity of maize to wd at the plant level (Lobell et al., 2014). These conclusions were also supported by the observation that selection signatures were observed in chromosome regions corresponding to plant architecture traits but not at those controlling physiological processes related to stress adaptation, suggesting that these traits have not been harnessed by breeding strategies (Welcker et al., 2022). Furthermore, drought‐tolerant maize hybrids developed via genetic engineering or marker‐assisted approaches were introduced in the market but essentially resulted in genotypes with higher yield in both well‐watered (ww) and dry conditions (Cooper et al., 2014; Nuccio et al., 2018; Sheoran et al., 2022), with perhaps the exception of ARGOS8‐modified maize (Shi et al., 2017).

Roots have a crucial role in water uptake; however, the exact ways variation of root system architecture and physiology affect drought tolerance remain elusive and are likely complicated by plant‐level trade‐offs involving the allocation and use of photosynthates and/or the response to other environmental conditions (Reynolds & Langridge, 2016; Tardieu & Tuberosa, 2010; Tardieu et al., 2018; Yu et al., 2024). Direct connection between root phenotypes and drought response was observed in a few cases: Fewer crown and lateral roots provided resources to increased axial growth, improving access to deeper and moister soil layers (Gao & Lynch, 2016; Zhan et al., 2015); reduced number of root cortex cells and increased root aerenchyma were shown to contribute positively to water‐stress tolerance likely by limiting plant respiratory costs (Klein et al., 2020; Schneider et al., 2023); and steeper root growth angle was associated with deeper roots (Klein et al., 2020; Lynch, 2018). However, the effect of root system biomass variation on shoot growth remains to be analyzed. In sorghum, root density was found higher than optimal for water uptake in many soil layers, suggesting that a lower investment in roots could be favorable in some circumstances (Robertson et al., 1993). A positive correlation between shoot and root biomasses was seen in wheat, but only up to a certain point, when additional increases in root biomass negatively impacted above‐ground biomass and grain yield, possibly due to an imbalance of resource allocation (Bektas et al., 2023).

Early vigor expressed as fast development of above ground vegetative biomass was recognized as a target for indirect selection for higher yield under drought in maize (Capo et al., 2023; Ruta et al., 2010; Trachsel et al., 2017, 2016). For instance, within a 2‐week maize maturity group, selection for early vigor and high stay green showed that early flowering hybrids could yield as much as later flowering hybrids (Trachsel et al., 2017). However, the combined effect of shoot early vigor and root system variation on drought tolerance remains cryptic, with possible constraints due to partially common genetic control and/or pleiotropy, physiological tradeoffs in resource allocations, and interactions with environment and management (Palta & Turner, 2019; Tardieu et al., 2018).

In this study, we aimed at testing the extent of genetic variation for early vigor and water balance traits, and their potential link with root growth, at the phenotypic and quantitative trait loci (QTL) levels. We used for that a maize introgression library (IL) population from an elite (B73) × landrace (Gaspé Flint) cross previously shown to segregate for major QTLs governing root system architecture (Salvi et al., 2016). The population was phenotyped in a high‐throughput phenotyping platform (Cabrera‐Bosquet et al., 2016) to evaluate water balance traits under water stress (wd) and non‐stress (ww) conditions, and with previously published results on seminal roots growth and architecture that we re‐analyzed in the context of this paper.

## 2 MATERIALS AND METHODS

### 2.1 Plant material and genomic characterization

A total of 69 lines from an IL population (Salvi et al., 2011) plus the two parents were tested. The maize reference line B73 was the IL recurrent parent while the donor parent was an accession of the early‐flowering North American landrace Gaspé Flint (hereafter named Gaspé). The IL molecular genetic characterization was refined using the 50K single nucleotide polymorphisms (SNP) Illumina Infinium array (Ganal et al., 2011). All marker positions are referred to the B73 RefGen_v2 as reported in Ganal et al. (2011). Monomorphic markers and/or with unknown or unclear physical map position on the maize reference genome and those with >10% of missing data were excluded which left a total of 18,860 SNPs (45.6%). Coverage of Gaspé genome provided by the IL collection is now higher (80.0%) than previously estimated based on SSR markers (66.9%; Salvi et al., 2011) with a mean of 3.3 Gaspé introgressions per line and an average length of approximately 39 Mb (equivalent to approximately 25 cM, on average). A total of 399 unique introgressed segments ranging from 0.04 to 64.20 Mb were identified, with an average of 3.67 Mb. A graphical genotype was constructed (Figure S1) representing consecutive introgressions with identical genotypic score at sequential SNPs and labeling the introgressed segments with their first SNP. Linkage disequilibrium (LD) between introgressed segments was evaluated using TASSEL 5 (Bradbury et al., 2007). LD *p* values were estimated by a two‐sided Fisher's exact test. Two introgressed segments were considered in high LD when the calculated *p* value was <0.01.

### 2.2 Experimental design and growth conditions

The phenotyping experiment was conducted at the PhenoArch plant phenotyping platform (Cabrera‐Bosquet et al., 2016) hosted at the M3P (Montpellier Plant Phenotyping Platforms, https://www6.montpellier.inrae.fr/lepse/Plateformes‐de‐phenotypage/Montpellier‐Plant‐Phenotyping‐Platforms‐M3P). Plants were grown in 9‐L cylindric (0.19 m wide and 0.40 m high) pots containing a 30:70 (v/v) mixture of a loamy soil and peat‐based compost. Eight replicates of each of the 69 IL lines plus the two parents were kept under ww (soil water potential Ψ > −1 bar/0.1 Mpa) conditions while additional eight replicates were subjected to mild wd conditions (soil water potential Ψ, ca. −0.4 Mpa). The water deprivation treatment was imposed when plants showed eight visible leaves. The desired soil moisture was reached gradually by imposing three progressively lower soil target moisture levels in order to avoid soil cracking and to allow for a homogeneous stress acclimation of the entire experimental population. Soil water content in pots was maintained at target values by daily watering of each pot using watering stations made up of weighting terminals with 1 g accuracy (ST‐Ex; Bizerba) and high‐precision pump‐watering stations (520U; Watson Marlow). The final wd target was kept constant until the end of the experiment (13.8 visible leaves on average). Air humidity, temperature and photosynthetically active radiation were monitored by eight sensors uniformly distributed within the greenhouse. Greenhouse temperature was maintained at 25 ± 3°C during the day and 18°C during the night. Supplemental light was provided either during day time when external solar radiation was below 300 W/m^2^ or to extend photoperiod using 400 W HPS Plantastar lamps (OSRAM) with 0.4 lamps/m^2^. Leaf temperature was continuously measured on eight B73 plants kept next to the experimental grid using thermocouples. Days were transformed for both ww and wd as 20°C equivalent days (thermal‐days, T‐days) as suggested in (Parent & Tardieu, 2012). Note that 1 T‐day is equivalent to the amount of heat accumulated in 24 h at 20°C constant temperature. All time‐related traits are reported as referred to T‐days, instead of calendar days. Daily RGB (red, green, blue) images of the plants (2056 × 2454 resolution) were captured from 13 views: 12 lateral views with a 30° rotational difference and one top view (for details, see Cabrera‐Bosquet et al., 2016). Plant pixels were separated from the background using thresholding algorithms and morphological operators implemented through OpenCV libraries (http://opencv.org). The pixel data were then converted into mm^2^ by calibrating the camera positions with reference objects. Total plant leaf area and fresh biomass were calculated daily using calibration curves developed from multiple linear regression models, which correlated processed images from the 13 views with leaf area measurements taken at various stages of growth (Brichet et al., 2017).

**Core Ideas**

- A maize introgression library was phenotyped for water use, water‐use efficiency, root and shoot growth.
- Quantitative trait loci (QTL) mapping identified seven major QTL clusters affecting shoot growth and water‐use efficiency.
- Gaspé Flint landrace confers early vigor QTL haplotypes with beneficial effect on water‐use efficiency.
- Reduced root‐to‐shoot ratio positively affects maize water‐use efficiency.

### 2.3 Traits collected in PhenoArch

Nine traits were collected in PhenoArch platform (Table 1). Most of the traits were collected in a temporal evaluation window starting from when the targeted stress level for the wd treatment was reached and ending at harvest. Shoot biomass accumulation (biomass) per plant was calculated as the biomass (g of fresh weight) increase between the start and the end of the evaluation window divided by the T‐days elapsed. Daily water use was estimated as the total amount of water (g) lost by transpiration during the evaluation window and its duration expressed in T‐day. WUE was estimated as the total shoot biomass (g of fresh weight) increase in the evaluation window divided by the total amount of transpired water (g) at the same time. Specific transpiration was calculated as the average amount of water (g) used between two phenotyping points and the average leaf area of the plant during the same interval. Biomass, transpiration, water use, and WUE were measured separately in ww and wd conditions. Early vigor was estimated as fresh shoot biomass before the water deprivation treatment at approximately eighth visible leaf stage. Leaf appearance rate approximates the number of leaves developed per T‐day and was estimated in ww condition only. For leaf appearance rate, the total number of completely developed leaves visible at the end of the experiment was first counted, followed by an additional score (from 0.3 to 0.8) in order to capture the partial development of the last visible leaf. A linear regression was fitted between leaf number and thermal time, and the slope of the regression was taken as leaf appearance rate. Biomass_res, water use_res, and transpiration_res represent the response of the three traits to wd and were calculated as the ratio between the standardized phenotypic values of each trait in ww and wd conditions.

**TABLE 1:** Description, range, mean value, and heritability (*h* ^2^) of the traits investigated in the 69 lines from a B73 × Gaspé introgression library population evaluated in well‐watered (ww) and water‐deficit (wd) conditions in high‐throughput phenotyping platform (PhenoArch) and selected root traits from Salvi et al. (2016) from a semi‐hydroponics (paper‐roll).

Description	Platform	Unit	Water regime	Min	Max	Mean	*h* ^2^
Biomass	PhenoArch	g/T‐day	wd	2.70	5.08	3.78	0.87
			ww	9.71	16.42	13.41	0.89
Transpiration	PhenoArch	g/m^2^/T‐day	wd	70.52	76.00	65.24	0.78
			ww	103.23	123.34	114.94	0.89
Water use	PhenoArch	g/T‐day	wd	93.95	126.72	110.75	0.86
			ww	186.24	278.44	242.87	0.93
Water‐use efficiency	PhenoArch	g/g	wd	2.85	4.24	3.5	0.88
			ww	4.09	7.19	5.62	0.88
Early vigor	PhenoArch	g fresh shoot biomass	–	18.48	91.82	48.32	0.92
Leaf appearance rate	PhenoArch	No. of leaves/20°C day	–	0.26	0.31	0.29	0.91
Biomass_res	PhenoArch	Biomass_ww/Biomass_wd	–	0.79	1.33	1.00	0.77
Transpiration_res	PhenoArch	Transp_ww/Transp_wd	–	0.88	1.51	1.02	0.88
Water use_res	PhenoArch	Water use_ww/Water use_wd	–	0.63	1.64	1.01	0.83
Embryonic root dry weight^a^	Paper‐roll	mg	–	9.4	40.0	26.1	0.81
Shoot dry weight^a^	Paper‐roll	mg	–	20.2	38.4	30.1	0.86
Root‐to‐shoot dry weight^b^	Paper‐roll	mg/mg	–	0.47	1.12	0.87	0.82

Abbreviations: res, response; transp., transpiration.

^a^ Reported from Salvi et al. (2016).

^b^ Computed here based on data from Salvi et al. (2016).

### 2.4 Traits collected in semi‐hydroponics (paper‐roll)

Trait analysis, correlation, and QTL analysis included three additional traits, namely embryonic root (primary and seminal roots formed during embryogenic phase) dry weight, shoot dry weight, and root‐to‐shoot ratio derived from a former experiment on the same maize population (Salvi et al., 2016) carried out at the seedling stage, with phenotypic data collected 9 days after germination in a semi‐hydroponic (paper‐roll) condition. Data for the first two traits are used as presented in Salvi et al. (2016), while root‐to‐shoot ratio was newly computed here as (embryonic root dry weight)/(shoot dry weight), mg/mg.

### 2.5 Microenvironmental effect estimation and correction

In order to evaluate and account for the effects of microenvironmental spatial variation, the following correction methods were evaluated: (i) no correction, (ii) mixed model with row and column effects on the experimental grid as fixed‐effect variate and genotype as random variate, (iii) moving average of deviations from population mean, (iv) moving average of deviations from the genotype mean, (v) both kinds of moving averages preceded by row and columns mixed model, and (vi) mixed model preceded by population and genotype deviations moving average. Outliers were detected as in Prado et al. (2019). For each trait, the correction method providing the highest broad sense heritability was adopted (Table S1).

### 2.6 Statistical analysis and QTL detection

Statistical analysis was performed using the software R (The R Core Team, 2016). Two‐tailed correlation tests were performed using the R package *psych* v. 1.6.7 and the *p* values corrected according to (Hochberg & Benjamini, 1990) for false discovery rate. After correcting each trait for the microenvironmental effects, as described above, we calculated best linear unbiased predictions (BLUPs) for each trait and line using the *lme4* package (Bates et al., 2014). We used the variable “Genotype” as the only random variable and excluded any other fixed‐effect variables. We applied the same method to compute BLUPs for traits measured in Salvi et al. (2016). Finally, we calculated correlation between traits measured in this experiment and those previously conducted on the same materials using the BLUP values. Principal component analysis (PCA) was performed on scaled values using the *princomp* function of the *stats* package (The R Core Team, 2016). Dunnett's multiple comparison test was carried out using the *multcomp* package (Hothorn et al., 2008). Broad sense heritability (*h* ^2^) was calculated using the *repeatability* function of the *repeatability* package (Wolak et al., 2012). Single introgression QTL analysis was carried out by *t*‐test comparisons between the lines carrying the given introgression and the lines without the same introgression and correcting the resulting *p* values according to the Bonferroni method. After having tested all the Gaspé introgressed genomic segments, a stepwise regression analysis was carried out to account for multiple introgressions in the same line. We herein define as QTL clusters those introgressions or groups of introgressions in strong LD (Fisher's test *p* < 0.01) that evidenced trait–genotype association (*p* value Bonferroni corrected < 0.01) for at least two traits. Genetically linked QTLs were considered as distinct in case of contrasting direction of genetic effect of the donor genomic fragment. In order to evaluate the putative impact of detected QTLs on traits related to grain yield and yield components, co‐localization with previously identified QTLs (Martinez et al., 2016; Millet et al., 2016) and meta‐QTLs (R. Li et al., 2024; Zhao et al., 2018), derived from integrated meta‐analysis of QTL data from different studies to identify a reduced confidence interval, in ww and wd conditions was evaluated as reported in Martinez et al. (2016).

### 2.7 Brace root analysis of selected lines

The number of brace roots and nodes with brace roots at adult stage were assessed in the field for three introgression lines (IL56, IL57, and IL64), each with a specific root QTL configuration (Section 3), and the B73 control. The experiment utilized single‐row plots (2.5‐m length, 11 plants/pot, 0.9‐m spacing) in a randomized complete block design with three replications at the University of Bologna Experimental Farm in Cadriano, Bologna. Contrasts were carried out using Dunnett's test with B73 serving as control using the *multcomp* package (The R Core Team, 2016). Plants were grown following local standard agronomic practices. Brace roots of four contiguous plants per plot were considered at flowering time, after manual plant excavation in the field, and both the number of brace roots per node and number of root‐carrying nodes were counted (Table S2).

## 3 RESULTS

### 3.1 Introgression lines reveal genetic variability in shoot and root growth, and water balance traits, with some outperforming B73 in WUE

**FIGURE 1:**
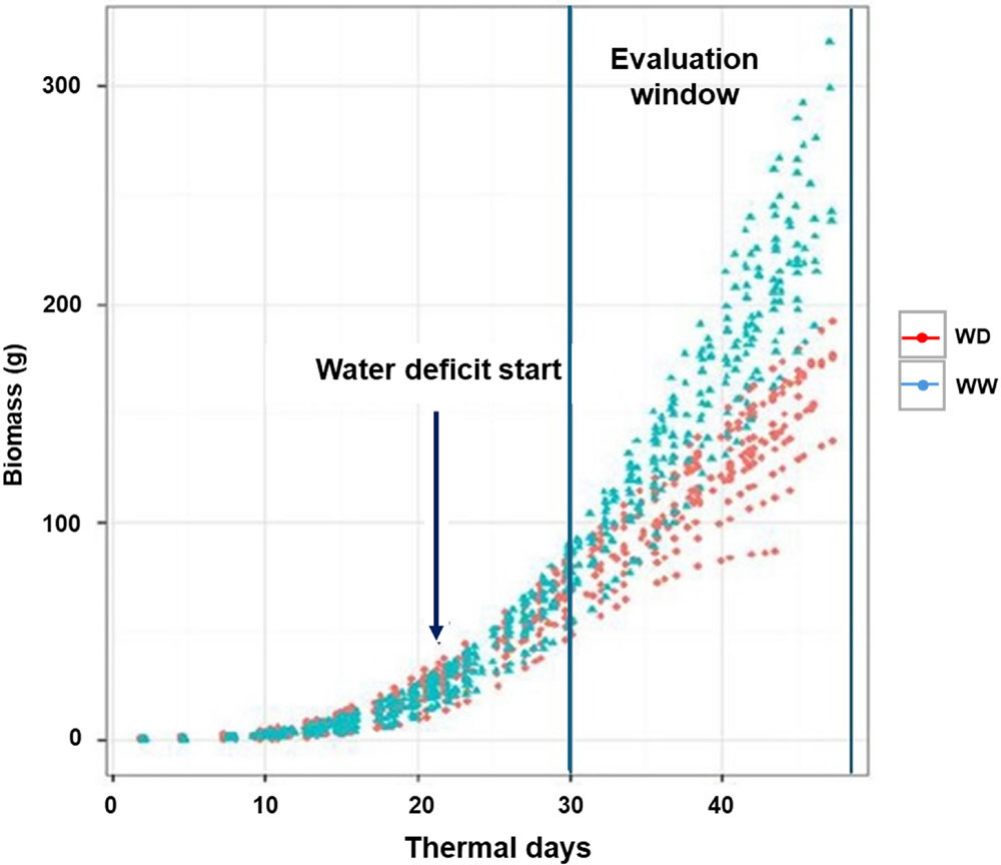
Biomass accumulation (BA) profiles, in g, in well‐watered (ww) and water‐deficit (wd) conditions during which homogeneous water conditions were maintained. The *x*‐axis represents thermal days (a thermal day is equivalent to the amount of heat accumulated in 24 h at 20°C constant temperature). Values for B73 only are shown as representative of the entire IL population. Each point represents a single BA estimate as detailed in Section 2, and each line of dots represents a single plant BA evolution throughout the experiment. Note that 10 plants in wd and 10 plants in ww were utilized. “Evaluation window” indicates the time period when trait values utilized for quantitative trait loci (QTL) analysis were collected.

Traits collected in platform (biomass, transpiration, water use, WUE, early vigor, leaf appearance rate, biomass_res, transpiration_res, water use_res) showed heritability values ranging from 0.77 to 0.92 (Table 1). Under wd conditions, B73 accumulated 3.55 g of biomass daily compared to 11.08 g in ww conditions (Figure 1). Considering the whole population, wd conditions significantly reduced biomass, transpiration, water use, and WUE by 69%, 42%, 46%, and 44%, respectively (*p* < 0.01; Table 1). Once the main effect of water treatment was considered, the genotypic effects explained a substantial portion of the variation for these traits with WUE showing the highest contribution (13.6% of total sum of squares) followed by biomass accumulation (5.2%). Water treatment × genotype interaction played a minor role, underlying that water use and transpiration are more prone to G × E interaction (0.8% and 1.0%, respectively) than biomass or WUE (1.6% and 3.0%, respectively).

**FIGURE 2:**
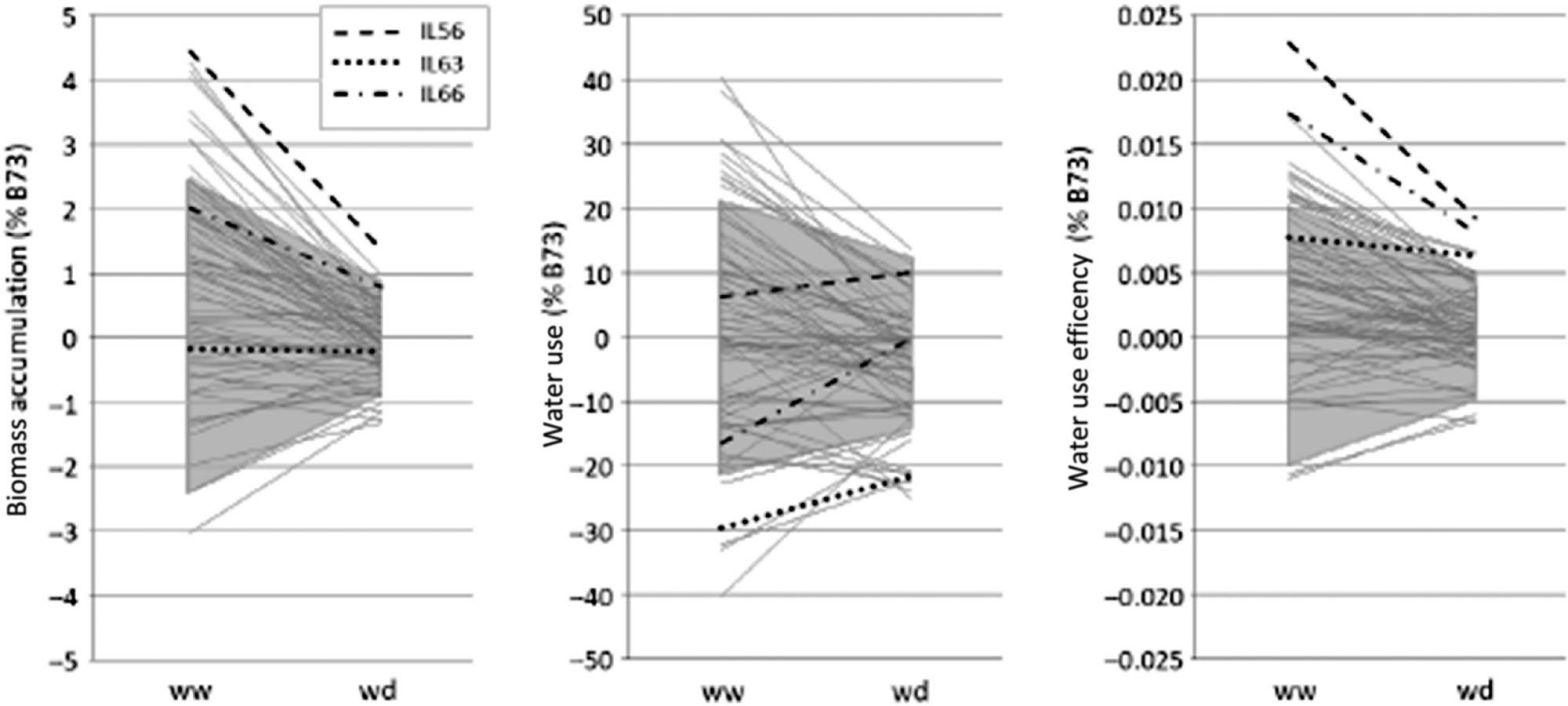
Reaction norms of the 69 introgression lines (B73 × Gaspé Flint) from well‐watered (ww) to water‐deficit (wd) conditions for biomass accumulation, water use, and water‐use efficiency (left to right, respectively). All trait values are represented as a mean proportional deviation from B73 (B73 = 0). Values included in gray‐shaded areas are not significantly different from B73 (Dunnett's test, threshold *p* = 0.01). Three lines (IL56, IL63, and IL66) showing interesting responses to wd conditions are highlighted (see text).

Performance of introgression lines for WUE and its components (biomass and water use), analyzed across the two water treatments and illustrated as reaction norms (Figure 2), revealed that 14 lines outperformed B73 in ww conditions, eight in wd, and five in both (*p *< 0.05, Dunnett's test vs. B73; Table S3; Figure 2). Lines with high WUE generally showed significantly higher biomass and similar or higher water use than B73. In both watering conditions, IL56 significantly outperformed B73 in terms of WUE and biomass accumulation with no increase in water use, while IL63 showed higher WUE than B73 in wd, resulting from a strong reduction in water use as well as no change in biomass accumulation rate. Interestingly, IL66 demonstrated the second highest WUE in both ww and wd conditions without extreme biomass or water use values. Lines characterized by low WUE showed significantly lower biomass in both ww and wd conditions (e.g., IL23, IL24, and IL54; Table S3).

Root traits (embryonic root dry weight, shoot dry weight, and root‐to‐shoot ratio) derived from a former experiment on the same maize population (Salvi et al., 2016) also showed high heritability values (*h* ^2^ = 0.81 − 0.86), which were similar to those collected for shoot traits (Table 1). Considerable range of phenotypic variation was observed for embryonic root dry weight (from 9.4 to 40.0 g, reference B73 line = 30.2 g; Table 1) and root‐to‐shoot ratio among introgression lines (from 0.47 to 1.12, reference B73 line = 0.87; Table 1). Across the entire population, six lines (IL03, IL47, IL48, IL49, IL56, and IL58) showed a lower root‐to‐shoot ratio than B73, while two lines (IL24 and IL 25) showed the opposite (Dunnett's test, *p* < 0.05; Table S3). Notably, four out of six lines (IL03, IL47, IL49, and IL56) with low root‐to‐shoot ratio in semi‐hydroponics exhibited higher biomass accumulation, three (IL03, IL47, and IL49) had higher water use values, and two (IL49 and IL56) showed higher WUE in ww condition in the PhenoArch platform experiment (Table S3). This advantage in terms of biomass accumulation and WUE was only maintained by IL56 in wd and largely lost by the other lines with low root‐to‐shoot ratio. Conversely, the only two lines (IL24 and IL25) with higher root‐to‐shoot ratio than B73 showed significantly lower water use in wd and one of which (IL24) also showed lower accumulation and WUE in wd besides a reduction in water use in ww conditions (Table S3).

Overall, these results support that chromosome introgressions of Gaspé Flint into the B73 genome determine a large genetic variation for both shoot and root growth and response to water use, transpiration, and WUE in both ww and wd conditions. Introgression lines characterized by low root‐to‐shoot ratio showed faster shoot biomass accumulation than B73 (despite the small size of the parent Gaspé plants), resulting in higher WUE.

### 3.2 WUE and related traits correlated negatively with root biomass and root‐to‐shoot ratio

**FIGURE 3:**
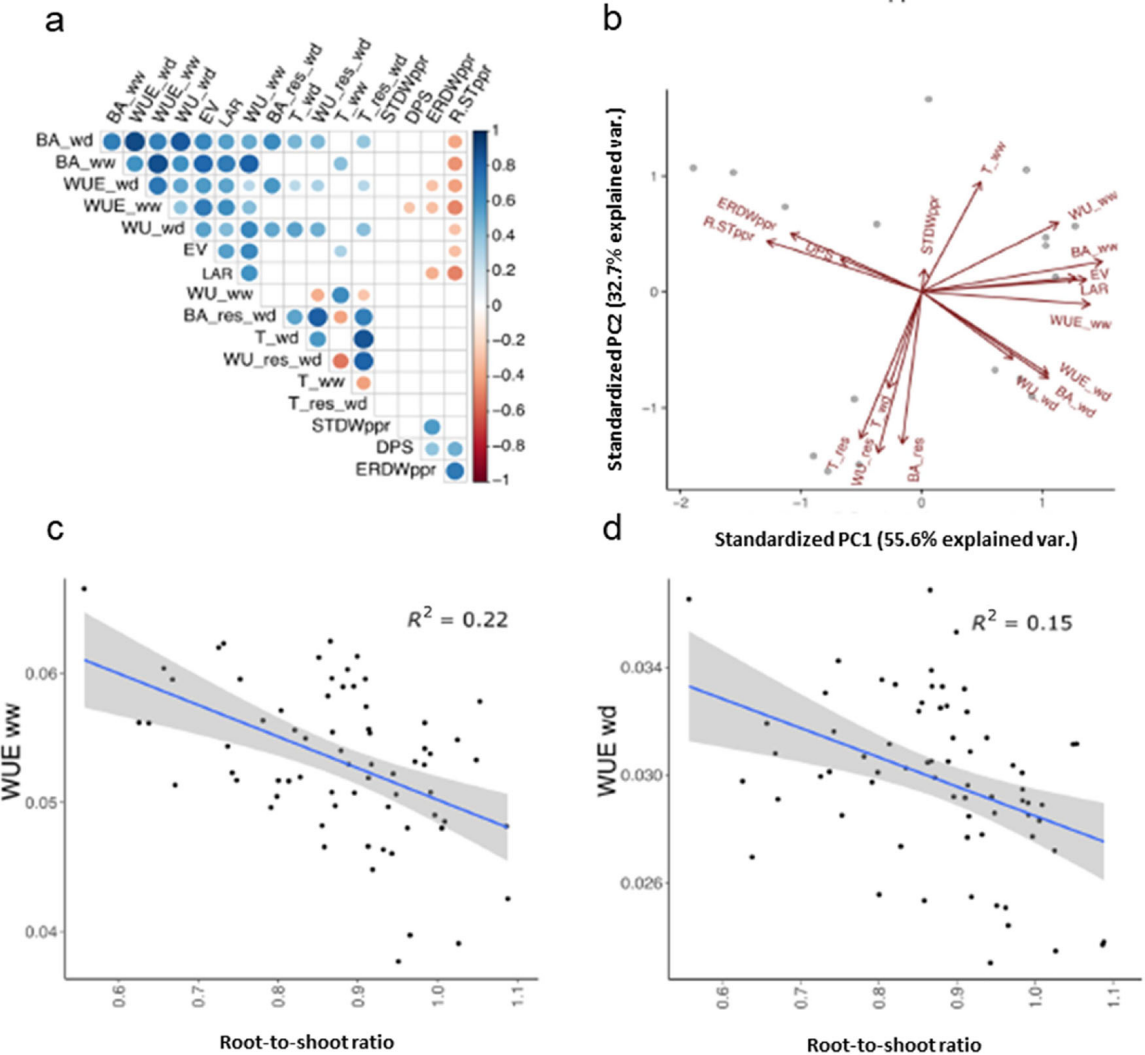
Variation and relationships among water balance traits in the two water treatments, well‐watered (ww) and water‐deficit (wd). (a) Pearson's correlations and (b) principal component analysis (PCA) for early vigor (EV), leaf appearance rate (LAR), biomass accumulation (BA), daily water use (WU), water‐use efficiency (WUE), specific transpiration (T), responses of biomass accumulation (BA_res), daily water use (WU_res), and transpiration (T_res) to wd condition (this paper), embryonic root dry weight (ERDWppr), shoot dry weight (STDWppr), and root‐to‐shoot ratio (R.STppr) at seedling stage in semi‐hydroponic paper rolls (from Salvi et al., 2016). (c and d) Correlations between root‐to‐shoot ratio in semi‐hydroponic paper rolls and WUE in ww and wd conditions, respectively.

Root‐to‐shoot ratio collected in semi‐hydroponics (original data from Salvi et al., 2016) correlated negatively with biomass accumulation and WUE collected in the platform in both ww and wd conditions (*r* ranging from −0.17 to −0.45; Figure 3a,c,d). Similarly, PCA showed, on its first axis, a contraposing effect between shoot growth traits on one hand and root‐to‐shoot ratio and embryonic root dry weight on the other hand (Figure 3b). Interestingly, response traits (transpiration_res, biomass accumulation_res, and water use_res) approximately aligned with the second (PC2) axis, appearing as independent from measured traits and with a partial positive correlation with biomass accumulation, transpiration, water use, and WUE measured in wd (Figure 3a,b). A strong correlation was observed between biomass accumulation and early vigor, which in turn positively correlated with WUE in both ww and wd conditions (Figure 3a,b). A significant positive correlation was observed between early vigor and leaf appearance rate (*r* = 0.58, *p* < 0.01); however, QTL analysis showed that this was mainly due to the presence of major QTL for the two traits on chromosome 3 (see Section 3.3). Overall, results of correlation analyses suggested that higher root‐to‐shoot ratio was not a favorable trait in terms of shoot biomass accumulation, early vigor, water use, and WUE.

### 3.3 QTL clusters and direction of genetic effect confirmed the influence of root trait variation on water balance traits

**FIGURE 4:**
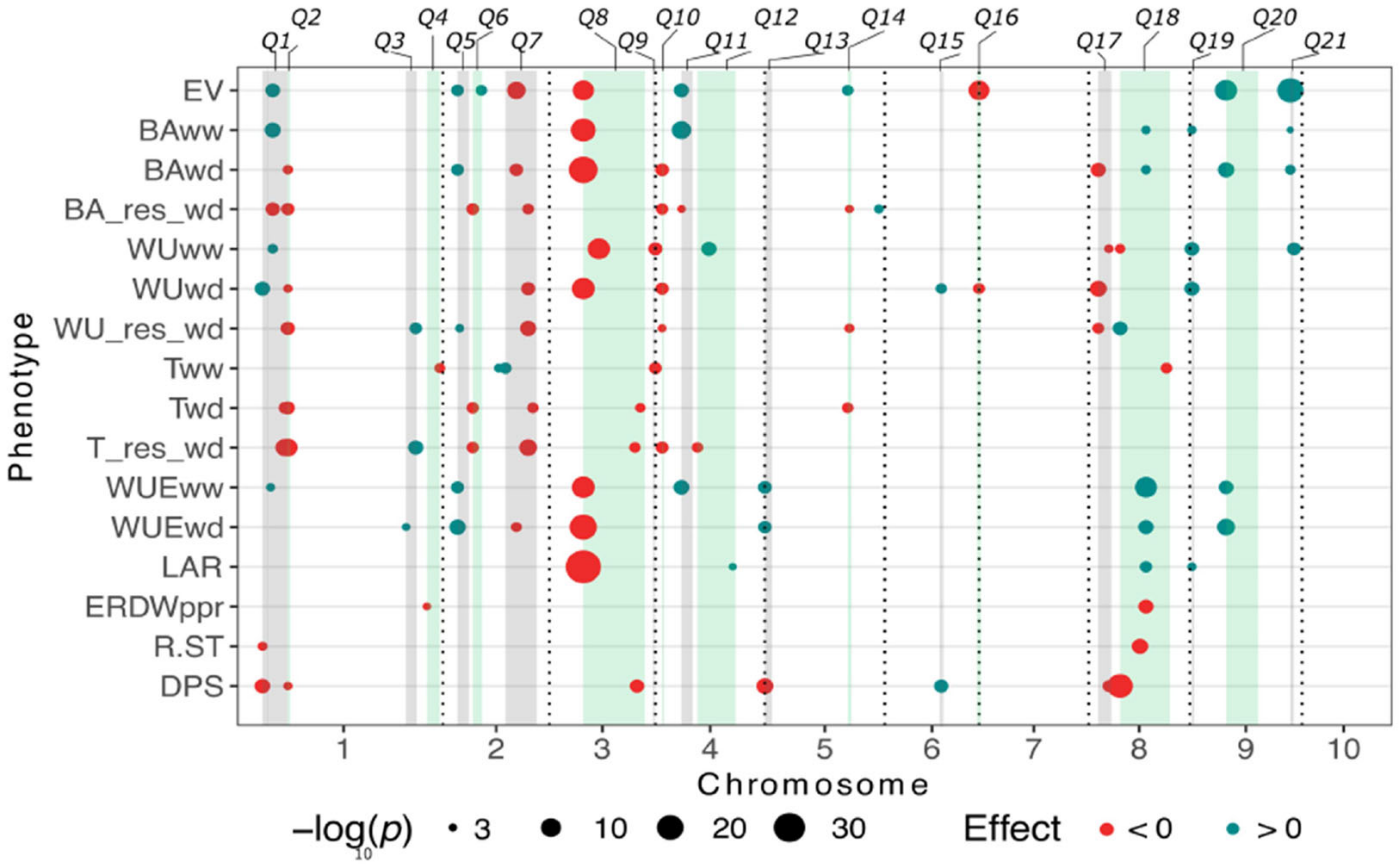
Quantitative trait loci (QTL) location for early vigor (EV), biomass accumulation (BA), daily water use (WU), water‐use efficiency (WUE), specific transpiration (T), BA, WU, and T responses to water‐deficit (wd) (BA_res_wd, WU_res_wd, T_res_wd), leaf appearance rate (LAR), embryonic root dry weight and root‐to‐shoot ratio at seedling stage in paper rolls (ERDWppr, R.STppr, data from Salvi et al., 2016), and days to male flowering (DPS, data from Salvi et al., 2011). Circles colocated with the QTL peaks and their diameter are proportional to the Bonferroni corrected −log_10_ *p* value; red and blue colors of the circles indicate negative and positive allelic effect of the Gaspé introgression, respectively. Shaded rectangles (green and gray) represent the confidence interval of the 21 QTL clusters.

QTLs for biomass accumulation, water use, and WUE‐related traits grouped in 21 QTL clusters, seven of which were particularly notable for their effects and/or number of controlled traits, namely *Q1* (chromosome 1), *Q5* and *Q7* (chromosome 2), *Q8* (chromosome 3), *Q18* (chromosome 8), and *Q20* and *Q21* (chromosome 9). At *Q1* (bins 1.01–1.03), the Gaspé allele increased biomass accumulation (+2.46 g/T‐day), water use (+21.16 g/T‐day), WUE (+0.99%), and early vigor (+6.3 g) in ww, and water use (+16.81 g/T‐day) in wd (Figure 4; Table S4), whereas affected biomass_res (−6.43%) and transpiration_res (−11.46%) negatively. Notably, the same region reduced root‐to‐shoot ratio at seedling stage (−24%). At *Q5* (bin 2.01–2.02) in the telomeric region of chromosome 2 short arm, the Gaspé introgression positively affected biomass accumulation (+0.76 g/T‐day, or +20.1%), water use_res (+8.85%), and WUE (+0.59%) in wd as well as WUE (+0.95%) in ww condition, and early vigor (+5.75 g, or +12%). Conversely, at *Q7* (bins 2.06–2.08), the Gaspé substitution had a negative early vigor effect (−6.89 g, or −14%), and a wd‐specific negative effect on biomass accumulation (−0.69 g/T‐day, or −18%), water use (−10.37 g/T‐day, or −9.4%), WUE (−0.38%), and transpiration (−15.65 g/m^2^), which negatively affected biomass_res (−5.26%), water use_res (−7.38%), and transpiration_res (−8.95%). At *Q8* (chromosome 3, bin 3.03–3.07), the Gaspé substitution strongly reduced biomass accumulation (−3.06 g/T‐day), water use (−38.15 g), and WUE (−1.12%) in ww conditions, and early vigor (−8.86 g or – 18%) and leaf appearance rate (−0.03 or −10.3%). A similar strong negative effect was observed for *Q8* in wd for biomass accumulation (−1.15 g/T‐day), water use (−15.03 g/T‐day), WUE (−0.69%), and transpiration (−6.78 g/m^2^). Accordingly, only transpiration_res was significantly affected by *Q8* (−6.51%). The Gaspé allele substitution at *Q11* (bin 4.03) had the strongest positive effect on biomass accumulation throughout the whole experiment (+3.97 g/T‐day) and was associated with a strong positive effect on early vigor (+7.9 g). These effects likely accounted for the positive effect on WUE in ww and the negative effect on biomass_res. Notably, *Q11* seemed to be effective under ww conditions only, with no effect detected in wd on any of the traits. At *Q18* (chromosome 8, bins 8.02–8.07), Gaspé substitutions positively affected WUE in both ww (+1.04%) and wd (+0.36%) as well as biomass accumulation in ww (+1.36 g/T‐day) and wd (0.46 g/T‐day). Similarly to *Q1*, Gaspé substitution at *Q18* region negatively affected root‐to‐shoot ratio at the seedling stage with the strongest negative effect (−24%) with a concurrent negative impact on embryonic root dry weight (−7.46 g). At *Q20* (bins 9.01–9.05), Gaspé alleles affected positively WUE in both water scenarios (+0.73% in ww and +0.45% in wd) and biomass accumulation in wd (+0.65 g/T‐day). The effect appears to be driven by the second strongest positive early vigor effect (+6.80 g, or +14.1%). Similarly, at *Q21* (bin 9.06), the strongest early vigor QTL (+19.08 g, or +39.5%) likely drove the positive effect on biomass accumulation both in wd (+1.22 g/T‐day, or 32%) and ww (+3.30 g/T‐day, or +25%) as well as on water use in ww (+29.72 g/T‐day, or +12%). Notably, *Q1* and *Q18* were the only two introgressed regions where the Gaspé allele simultaneously lowered root‐to‐shoot ratio while increasing WUE.

In order to test whether *Q1* and *Q18* also affect root development at the adult phase, three introgression lines (IL56, IL57, and IL64) identified to carry Gaspé introgressions encompassing *Q1* and *Q18* or both (Figure S1) were analyzed at the adult stage in a field experiment. Lines IL56 and IL57 with *Q18* or *Q18* + *Q1* introgressions, respectively (Figure S1), showed a lower number of brace roots in the field as compared to B73, with the maximum effect observed for the line carrying the double introgression (11.1 roots in IL57 vs. 17.9 roots in B73, *p* < 0.001; Table S2). In addition, the number of nodes with brace roots was significantly lower in IL57 as compared to B73 (1.88 nodes in IL57 vs. 2.60 nodes in B73, *p* < 0.05; Table S2). Results were in the same direction but not significant for IL64, which only carries the *Q1* introgression (16.1 roots in IL64 vs. 17.9 in B73, and 2.50 nodes in IL64 vs. 2.60 in B73, not significant).

Overall, biomass accumulation, WUE, and early vigor were affected by 14, 9, and 10 QTL clusters out of 21, respectively. On the other hand, only two QTL clusters had an effect on root‐to‐shoot ratio (Table S4).

### 3.4 Water balance QTL clusters overlapped with known grain yield QTLs

Overlaps between QTLs for water balance and root traits were searched based on a dataset of 856 QTLs assembled from previously published data (Martinez et al., 2016; Millet et al., 2016; see Tables S5 and S6) and on 119 meta‐QTLs (R. Li et al., 2024; Zhao et al., 2018; see Tables S5 and S6) related to grain yield (plant height, ear height, anthesis‐silking interval, ear weight, cob weight, 100‐kernel weight, and ear length) under ww and/or wd conditions. Clusters *Q1*, *Q3*, *Q5*, *Q6*, *Q7*, *Q8*, *Q12*, *Q14*, *Q15*, *Q18*, *Q20*, and *Q21* showed overlapping intervals in both QTLs retrieved from Martinez et al. (2016) and Millet et al. (2016) as well as in meta‐QTLs from Zhao et al. (2018) and R. Li et al. (2024). On the other hand, *Q11*, *Q13*, *Q16*, *Q17*, and *Q19* colocated with 12 QTLs but no meta‐QTLs were reported in this region, and *Q10* cluster showed overlapping intervals only with meta‐QTLs from R. Li et al. (2024). A notable overlap was identified at chromosome 1 (bins 1.01–1.03), where 44 yield QTLs and four meta‐QTLs overlapped with *Q1*, a QTL cluster of QTLs for water balance and root‐to‐shoot dry weight ratio traits. As shown here and in a former experiment (Salvi et al., 2016), Gaspé allele at *Q1* cluster reduces root number decreasing root‐to‐shoot dry weight ratio. As shown in this experiment, this likely promotes early vigor and WUE, with a possible more general effect on yield components (KW and KN). A notable number of QTL overlaps also occurred at chromosomes 2, 3, 8, and 9 for *Q7*, *Q8*, *Q18*, and *Q20* clusters, respectively. Gaspé allele at *Q7* cluster (bins 2.05–2.08), which is negatively associated with water balance traits in wd conditions and with a positive effect on transpiration rate in ww conditions, is colocated with 42 QTLs and four meta‐QTLs. The *Q8* cluster (bins 3.03–3.07) showed the highest number of overlapping QTLs (80) and overlapped with four meta‐QTLs. Despite the Gaspé allele at this cluster negatively affects early vigor, biomass accumulation, and water balance related traits in both ww and wd conditions, it promotes early flowering favoring drought escape. The *Q18* cluster (bins 8.02–8.07), with an effect on WUE and biomass accumulation in ww and wd conditions, overlapped with 70 QTLs and with eight meta‐QTLs. Notably, many QTLs for root traits map within *Q18* cluster along with the flowering time QTL *Vgt1* (Salvi et al., 2007). Similar to *Q1* cluster, *Q18* decreases root‐to‐shoot ratio showing a stronger effect in reducing both seminal and brace roots (Tables S2 and S4). Moreover, 49 QTLs and seven meta‐QTLs overlapped with *Q20* cluster (bins 9.01, 9.03, and 9.05) that showed, along with *Q21*, the strongest effect on early vigor strongly correlated to WUE and biomass accumulation in both ww and wd conditions. No overlapping QTLs or meta‐QTLs were found in *Q2* and *Q9* clusters. Notably, at *Q19* cluster (bin 9.01), the Gaspé introgression showed the strongest positive effect on water use traits in both ww and wd conditions. However, the low genetic resolution caused by long Gaspé introgressions, and the renowned effects of major phenology QTLs mapped herein, prevents further speculation on their functional overlap with yield QTLs.

## 4 DISCUSSION

### 4.1 WUE at adult vegetative stage is negatively associated with root‐to‐shoot ratio

A parsimonious root system in terms of reduced carbon allocation affecting architecture, size, and/or inner anatomy has been repeatedly suggested as advantageous for water capture and uptake (Hostetler et al., 2023; Lynch, 2018; Maqbool et al., 2022; van Oosterom et al., 2016). Our study revealed a negative correlation between (i) root‐to‐shoot ratio and embryonic root dry weight and (ii) shoot biomass, water use, and WUE collected in platform at adult vegetative stage in ww conditions in line with the parsimonious hypothesis. The negative correlation was also supported by ILs performance and QTL positions for the same traits. Specifically, four of six ILs with root‐to‐shoot ratio lower than B73 in semi‐hydroponics showed better water balance traits than B73 when grown in platform. Based on a former study, we know that a major component of the difference in root‐to‐shoot ratio in this maize population is due to genetically controlled difference in number of seminal roots (Salvi et al., 2016). Root‐to‐shoot ratio and WUE QTLs were co‐mapped at Q1 and Q18, with Gaspé allele decreasing root‐to‐shoot ratio while increasing WUE in both cases. Q1 confidence interval includes *Rtcs*, a gene previously characterized for its role in seminal and crown root development (Hochholdinger et al., 2018; Taramino et al., 2007), and suggested as a candidate for major QTLs shown to affect root system architecture at the juvenile stage in a B73 × Mo17 background (Bohn et al., 2006) and also for a QTL for number of seminal roots (Gaspé contributing the allele reducing root number) in this population (Salvi et al., 2016). Similarly, the chromosome bin 8.05 in Q18 is a hotspot for root QTLs (Burton et al., 2014; Pestsova et al., 2016; Zurek et al., 2015). Notably, *Rum1*, a maize gene involved in seminal and lateral roots development (Baer et al., 2023; Woll et al., 2005), also maps within Q18 confidence interval. We speculate that the advantage was provided by the reduced root‐to‐shoot ratio driven by a reduction in axial root number (in this case, seminal root, crown, and early brace roots), as shown by the QTLs for number of seminal roots mapped in the same chromosome regions by Salvi et al. (2016) and by the field test experiment in this study, where IL lines carrying Gaspé introgressions at the Q1 and Q18 indeed showed fewer brace roots than the control. Thus, the reduced root‐to‐shoot ratio genetically promoted by Gaspé chromosome introgressions at these QTLs likely enabled the allocation of additional carbon resources to shoot growth with a final positive effect on WUE.

At the same time, the relatively smaller root system did not hinder the water uptake capacity given the non‐limiting water availability conditions of the ww treatment in this experiment and the known flexibility in water uptake capacity of both embryonic and adult maize roots (Protto et al., 2024; Rishmawi et al., 2023). Accordingly, Guo and York (2019) proved that artificial excision of part of the axial root system in maize might benefit shoot development and drought tolerance. In wd conditions, the effects of Q1 and Q18 on water balance traits were substantially different, with the Q18 introgression maintaining its beneficial effect on water balance traits while Q1 introgression negatively affected transpiration and water‐use responses. A possible reason of this discrepancy might be due to different effects of the two genomic regions on nodal root differentiation, with Gaspé allele at Q18 showing a much stronger effect than at Q1 in reducing both seminal and brace root number as also demonstrated by testing the relevant lines (IL56, IL57, and IL64) in the field (Table S2), thus, allowing the allocation of a higher portion of photosynthates to shoot growth in later growth stages.

### 4.2 QTLs for early vigor and water balance traits

In our study, early vigor measured as fresh shoot biomass before the water deprivation treatment in platform was one of the traits positively correlated with water use and WUE in both water regimes, although correlation was higher in ww (Figure 3a,b). This is an expected finding since early‐vigor genotypes develop larger canopies earlier, which in turn can better sustain plant growth (Tardieu et al., 2018). In wd conditions, the beneficial effect of early vigor QTLs on other WUE traits was recorded for *Q1*, *Q5*, *Q20*, and *Q21*. The Gaspé introgression at *Q20* and *Q21* had the strongest effect. *Q20* might be of particular agronomic interest given the high number (56 in total) of co‐mapping grain yield QTLs based on published information (Tables S5 and S6). The *Q20* confidence interval also includes *VPP1*, a vacuolar‐type H+‐pyrophosphatase affecting photosynthetic efficiency and root development (Wang et al., 2016), *PTF1*, a bHLH transcription factor that regulates root growth and abscisic acid synthesis (Z. Li et al., 2019), and *TIP1*, encoding a S‐acyltransferase that affects root hairs elongation (Zhang et al., 2020), all possibly contributing to the establishment and growth of the maize seedling in the early phases. At *Q1*, the Gaspé allele, besides reducing the root‐to‐shoot ratio, also increased early vigor, with a strong positive effect on WUE. It was shown that *LBD2* gene mapping within *Q1* interval influence plant germination rate, chlorophyll content, and fresh weight, thus, contributing to enhance early vigor in maize (Jiao et al., 2022). As conceivable, the reduction in root‐to‐shoot ratio (in turn due to a major QTL for the number of axial roots) was the underlying main factor providing more carbon resources to sustain higher shoot growth and, thus, early vigor.

## 5 CONCLUSIONS

Besides being a well‐known source of early flowering and root architecture alleles, the early‐flowering maize landrace Gaspé Flint was found in this study to be a valuable source of early vigor QTL haplotypes with beneficial effects on WUE. We report for the first time in maize a sizeable negative correlation between root‐to‐shoot ratio at the seedling stage and shoot biomass accumulation, and water use and WUE at adult vegetative stage. This correlation was clear in ww conditions and not in wd. Additionally, we show that variation for root‐to‐shoot ratio was under the control of major QTLs for number of seminal roots at two chromosomal regions on chromosomes 1 and 8 harboring root development candidate genes, likely driving biomass partitioning, early vigor, and eventually WUE. At least under ww conditions, a reduction of number of seminal roots was associated to stronger early vigor and higher WUE. This study provides clear evidence of the value and effectiveness of high‐throughput phenomic investigations for the genetic dissection of physiological processes of agronomic impact such as plant response to wd. Additionally, our results provide an opportunity for marker‐assisted selection and better leveraging the maize QTLome (Salvi & Tuberosa, 2015) aimed at fine modeling root system architecture to specific target environments and, once the relevant QTL will eventually be cloned, to identify native beneficial alleles and/or design more effective ones through gene editing.

## AUTHOR CONTRIBUTIONS

**Giuseppe Sciara**: Formal analysis; investigation; software; writing—original draft; writing—review and editing. **Francesco Camerlengo**: Formal analysis; investigation; software; writing—original draft; writing—review and editing. **Claude Welcker**: Conceptualization; project administration; supervision; writing—original draft; writing—review and editing. **Llorenç Cabrera‐Bosquet**: Data curation; investigation; methodology; software. **Antonin Grau**: Investigation; software. **Maria Angela Cané**: Investigation. **Riccardo Bovina**: Investigation. **Francois Tardieu**: Conceptualization; project administration; resources; writing—original draft; writing—review and editing. **Roberto Tuberosa**: Conceptualization; project administration; resources; writing—original draft; writing—review and editing. **Silvio Salvi**: Conceptualization; project administration; supervision; writing—original draft; writing—review and editing.

## CONFLICT OF INTEREST STATEMENT

The authors declare no conflicts of interest.

## Supporting information



Supplementary Figure S1. Graphical genotype of the Gaspé Flint introgression library.

Supplementary Table S1. Broad‐sense heritability of each trait.

Supplementary Table S2. Number of brace roots and brace root nodes of the introgression lines with low root‐to‐shoot ratio and B73.

Supplementary Table S3. Dunnet's test of the comparisons between the introgression library and B73.

Supplementary Table S4. QTLs table.
Supplementary Table S5. QTLs overlaps.
Supplementary Table S6. Traits considered for yield.

## Data Availability

All data supporting the findings of this study are available within the paper and its Supporting Information.
